# Structure-based identification of galectin-1 selective modulators in dietary food polyphenols: a pharmacoinformatics approach

**DOI:** 10.1007/s11030-021-10297-1

**Published:** 2021-09-05

**Authors:** Shovonlal Bhowmick, Achintya Saha, Nora Abdullah AlFaris, Jozaa Zaidan ALTamimi, Zeid A. ALOthman, Tahany Saleh Aldayel, Saikh Mohammad Wabaidur, Md Ataul Islam

**Affiliations:** 1 Department of Chemical Technology, University of Calcutta, 92, A.P.C. Road, Kolkata, 700009 India; 2 Nutrition and Food Science, Department of Physical Sport Science, Princess Nourah Bint Abdulrahman University, P.O. Box 84428, Riyadh, 11671 Saudi Arabia; 3 Department of Chemistry, College of Science, King Saud University, P.O. Box 2455, Riyadh, 11451 Saudi Arabia; 4 Division of Pharmacy and Optometry, School of Health Sciences, Faculty of Biology, Medicine and Health, University of Manchester, Oxford Road, Manchester, M13 9PL UK; 5 Department of Chemical Pathology, Faculty of Health Sciences, University of Pretoria and National Health Laboratory Service Tshwane Academic Division, Pretoria, South Africa

**Keywords:** Galectin-1, Dietary polyphenols, Molecular docking, Molecular dynamics, MM–GBSA

## Abstract

**Abstract:**

In this study, a set of dietary polyphenols was comprehensively studied for the selective identification of the potential inhibitors/modulators for galectin-1. Galectin-1 is a potent prognostic indicator of tumor progression and a highly regarded therapeutic target for various pathological conditions. This indicator is composed of a highly conserved carbohydrate recognition domain (CRD) that accounts for the binding affinity of β-galactosides. Although some small molecules have been identified as galectin-1 inhibitors/modulators, there are limited studies on the identification of novel compounds against this attractive therapeutic target. The extensive computational techniques include potential drug binding site recognition on galectin-1, binding affinity predictions of ~ 500 polyphenols, molecular docking, and dynamic simulations of galectin-1 with selective dietary polyphenol modulators, followed by the estimation of binding free energy for the identification of dietary polyphenol-based galectin-1 modulators. Initially, a deep neural network-based algorithm was utilized for the prediction of the druggable binding site and binding affinity. Thereafter, the intermolecular interactions of the polyphenol compounds with galectin-1 were critically explored through the extra-precision docking technique. Further, the stability of the interaction was evaluated through the conventional atomistic 100 ns dynamic simulation study. The docking analyses indicated the high interaction affinity of different amino acids at the CRD region of galectin-1 with the proposed five polyphenols. Strong and consistent interaction stability was suggested from the simulation trajectories of the selected dietary polyphenol under the dynamic conditions. Also, the conserved residue (His44, Asn46, Arg48, Val59, Asn61, Trp68, Glu71, and Arg73) associations suggest high affinity and selectivity of polyphenols toward galectin-1 protein.

**Graphic Abstract:**

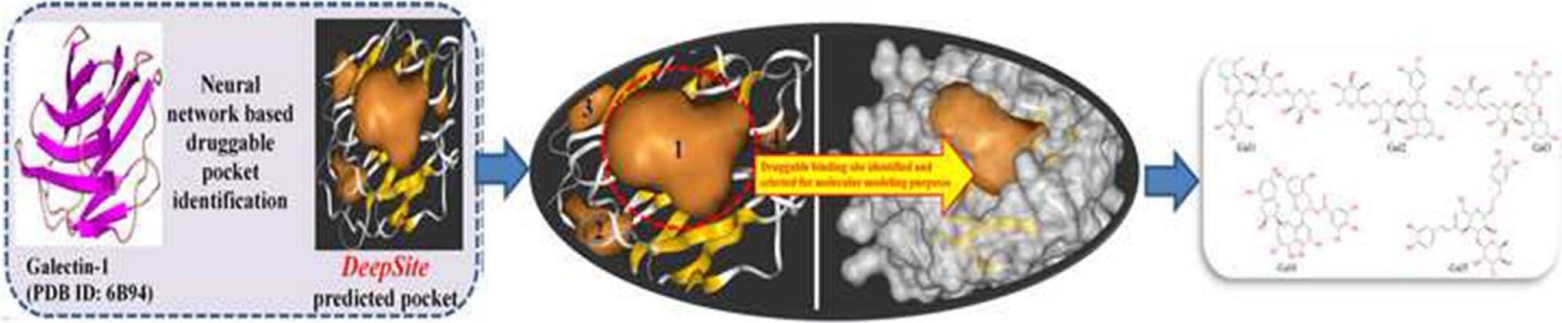

## Introduction

Over the last few years, several studies indicate the emergence of the family of galectins as interesting drug targets for various pathophysiological conditions [1–16]. Galectins are a conserved family of lectins, which are formally known as S-type or S-Lac lectins [17, 18]. These lectins are phylogenetically conserved and present in different animal species with a wide taxonomic distribution [19]. Almost 40 years ago, the first member of this protein family was identified, which was initially known by various names, such as electrolectin, β-galactoside-binding lectin, or galaptin. The systematization of the nomenclature for galectins renamed the family as galectin-1 [20, 21]. Currently, there are almost 15 mammalian galectins, which are structurally characterized as prototype (viz*.* galectin-1, –2, –5, –7, –10, –11, –13, –14, –15), chimera (i.e., galectin-3), and tandem repeat (viz. galectin–4, –6, –8, –9, –12) [22–24]. These three groups of galectins possess a well-defined carbohydrate recognition domain (CRD) constituting highly evolutionary conserved amino acid sequences and a β-sandwich structure that confers their ability to bind with β-galactoside-rich glycoconjugates. Galectins are found inside (intracellular) as well as outside the cells (extracellular) to control various cellular or regulatory programs [25]. Moreover, galectins are expressed in a variety of cells. Although they are synthesized or found in the cytosol, galactins can also be translocated in the nucleus. However, the mechanism involved in the secretion of these proteins on the extracellular compartments is still poorly elucidated [26]. The family of galectins is involved in a wide range of biological processes, such as cellular growth regulation, cell transformation, adhesion/migration, immunoregulation, chemotaxis and angiogenesis, invasion and metastasis, immune escape, and various key aspects of carcinogenesis-associated implications and other medical applications [23, 27]. Moreover, these proteins are deeply involved in a variety of biological activities, such as the recognition and destruction of pathogens to alleviate their entry into the host cells [19, 28–30]. Precisely, galectins act as subtle intermediates to decipher the information of the host immune cells and microbial structures during microbial infection contained within the glycan and thereby modulate various types of cell-to-cell communication [31].

There have been substantial insights into the biological function and properties of specific galectins in the last few decades. Among all the members of the galectin family, the prototype member galectin-1 protein has been considered as a modulator of cell migration and tissue invasion [32, 33]. Hence, galectin-1 protein is considered a prognostic indicator for tumor progression in pathological analyses and a target for glycocluster design [34]. In addition, human galectin-1 acts as a multifunctional effector that participates in various protein–carbohydrate and protein–protein or protein–lipid interactions [35]. Galectin-1 protein has been recognized to play a key role in the immune system by interceding the apoptosis of the activated T cells with CD7 [36]. The high expression of galectin-1 protein has direct medical implications on the development of tumorigenesis [37, 38]. Several studies have reported the significant influence of high or increased expression of the galectin-1 protein on different human cancers, including breast cancer [39], head and neck cancer [40], renal cell carcinoma or kidney cancer [41], lung cancer [42], ovarian cancer [43], thyroid carcinoma [44, 45], uterine adenocarcinoma [46], prostate cancer [47] and pancreatic cancer [48]. Owing to the overexpression of galectin-1 in different cancer cell lines, the protein has been proposed as a potential target for therapeutic intervention and a predictive diagnostic marker [26, 49].

The galectin-1 protein consists of ∼135 amino acids and a conserved CRD motif specifically attributed to the carbohydrate-binding groove, which is long enough to accommodate several saccharide derivatives. Similar to saccharides, polyphenols are a large heterogeneous group of phytochemicals founds in plant-based foods with potentially positive effects on human health [50]. In addition, dietary polyphenols are known to be safe with remarkable nutritional benefits, including antioxidant property that is beneficial against oxidative stress-related diseases, such as cancer, aging, and cardiovascular diseases. Also, dietary polyphenols play a significant role in several other activities, including anti-inflammatory, anti-proliferative, anti-atherosclerosis, neuro-protective, anti-diabetic, anti-mutagenic, antimicrobial, and hepato-protective actions [51]. The epidemiological evidence on polyphenol-rich diets has suggested the significant impact of the activity of the phenolic compounds on the modulation of intracellular signaling pathways and gene expression [52, 53]. Moreover, dietary polyphenols might influence carbohydrate metabolism at different levels [53]. Polyphenols can directly interact with proteins by the creation of either hydrophobic or hydrophilic interactions and result in the formation of soluble or insoluble complexes [50, 54], leading to the denaturation of the enzymatic protein chain or enzymatic inhibition. The interactions between various polyphenols and proteins/enzymes viz. monoamine oxidase, phospholipase A2, Kelch-like ECH-associated protein 1, cytochrome P450, nuclear factor kappa B, laminin receptor, metalloproteinases, extracellular microbial enzymes, 3-hydroxy-3-methylglutaryl coenzyme A reductase, hepatitis C virus NS3 protease, cholera toxin, virus-encoded integrase, α-glucosidase, retroviral reverse transcriptase, enterotoxin, leukotoxin, α-amylase, pro-oxidant enzymes, β- and γ-secretases, etc. have been reported to have significant health benefits [51]. Despite considerable research, there has been significant progress toward understanding the functions and inhibition mechanisms of galectins with respect to cancer. However, there have been no significant studies on the selective inhibitory mechanisms of dietary polyphenols against galectin-1 in promoting the anticancer therapeutic strategies, particularly using exhaustive molecular modeling techniques. Therefore, various dietary polyphenols extracted from the Phenol-Explorer database (http://phenol-explorer.eu/) were extensively analyzed in this study using many advanced computational techniques for modulating the galectin-1 protein by means of effective interactions in the CRD region. In particular, the exhaustive computational approaches combining the machine-learning-based recognition of the ligand binding site on the galectin-1 protein, absolute binding affinity predictions between the dietary polyphenols and galectin-1, extra-precision molecular docking, and all atomistic long-range 100 ns molecular dynamics (MD) simulation studies, and molecular mechanics–generalized born surface area (MM–GBSA)-based binding free energy estimation were implemented for identifying the potential polyphenols to be used as galectin-1 inhibitors/modulators.

### Materials and methods

**Fig. 1:**
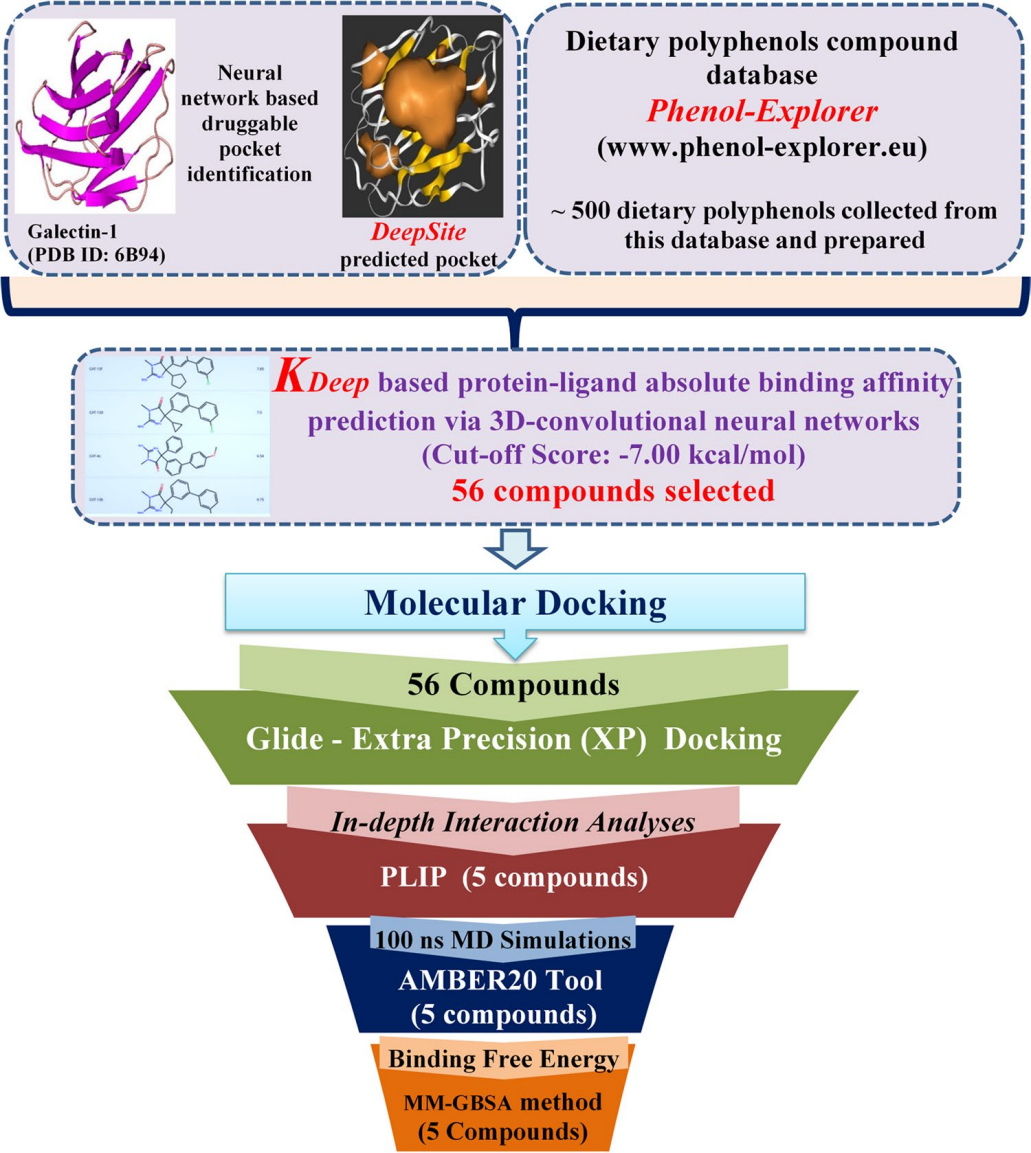
Schematic workflow for the identification of promising inhibitors/modulators of galectin-1 protein from dietary polyphenols

The complete workflow for the identification of the potential dietary polyphenol-based inhibitors/modulators of galectin-1 protein investigated in the current study is depicted in Fig. 1.

### Collection and preparation of dietary polyphenols

About 500 dietary polyphenol compounds were collected from the Phenol-Explorer database available at www.phenol-explorer.eu [55]. The Phenol-Explorer database is the first free user-friendly web interface available for the research communities, which consists of several groups of bioactive dietary polyphenols (such as lignans, flavonoids, phenolic acids, and stilbenes), along with their retention factors. All the polyphenol compounds were manually verified for any structural or valance errors before employing them for modeling purposes. Further, the preparation of all the dietary polyphenols was carried out by employing the ‘LigPrep’ module [56] in the Maestro interface of the Schrödinger suite. Using the default setting in the ‘LigPrep’ module, the most probable three-dimensional (3D) low-energy conformers for each polyphenol were generated. Also, the ionization states were achieved in the pH range of 7.0 ± 2.0 for all the polyphenols. During the optimization phase of each polyphenol structure, the optimized potentials for liquid simulation (OPLS3) force field [57] were applied for further analysis.

### Preparation of the target protein and prediction of the drug binding sites in galectin-1

The 3D crystal structure of the galectin-1 protein was obtained from the Protein Data Bank (PDB) [58], namely PDB ID: 6B94 [59], with a resolution of 1.80 Å and observed R-value of 0.21. After retrieving the crystal structure, the ‘Protein Preparation Wizard’ [60] utility tool of the Schrödinger suite in the Maestro interface was employed appropriately to rectify any conformational errors in the structure, as described in the literature [61–68]. Using the standard protocol of the ‘Protein Preparation Wizard,’ the galectin-1 crystal structure was pre-processed to achieve the lowest energy minimized geometry conformation for the selected protein. The prepared galectin-1 structure was used for subsequent molecular modeling studies.

Among the different computational methods [69] used for the prediction of the protein–ligand binding site, the systematic introduction of the machine or deep learning algorithms is one of the most popular techniques that are tremendously used in the SBDD pipeline [70, 71]. In the current study, DeepSite—a 3D deep convolutional neural network (DCNN)-based protein binding site predictor tool [72] was employed to predict the possible drug binding sites on the galectin-1 protein. The DeepSite tool is freely accessible at www.playmolecule.org domain. In particular, DeepSite is a complete machine-learning-based technique employed to infer information of the binding site characteristics of the protein using the deep library of more than 7000 proteins in the scPDB database [73]. Therefore, the prepared protein structure was browsed in the web application of DeepSite for the prediction of the protein-binding pockets based on the application of deep neural networks.

### The absolute protein–ligand binding affinity and energy prediction using the K_DEEP_: 3D convolutional neural network method

*K*_*DEEP*_ is a state-of-the-art 3D convolutional neural network-based protein–ligand binding affinity predictor tool [74] implemented in this study to deduce the absolute binding affinity of each dietary polyphenol toward the galectin-1 protein. The *K*_*DEEP*_ tool is based on the DCNNs model, which has been pre-trained, tested, and validated using the PDBbind v.2016 database. In order to perform the *K*_*DEEP*_ utility program, two input files were added for the prepared galectin-1 protein and all the dietary polyphenols. The other input features were used as default in the web application available at https://www.playmolecule.com/Kdeep/. Initially, *K*_*DEEP*_ gives the 3D voxel representation of the binding site by considering eight different pharmacophoric-like features/descriptors (such as hydrogen bond donor or acceptor, aromatic, hydrophobic, metallic, positive or negative ionizable, and total excluded volume) of the protein and ligands. The descriptors are further used for model generation by applying the algorithm of the 3D CNN model, which involves the study of the binding affinity and, thus, predicts the absolute binding affinity. Herein, the *K*_*DEEP*_ tool, a faster machine-learning approach, was employed for the prediction of the binding affinity between galectin-1 protein and dietary polyphenol ligands for subsequent analyses by taking a theoretical binding energy cutoff value of –7.00 kcal/mol.

### Docking grid preparation and execution of the Glide XP docking

**Fig. 2:**
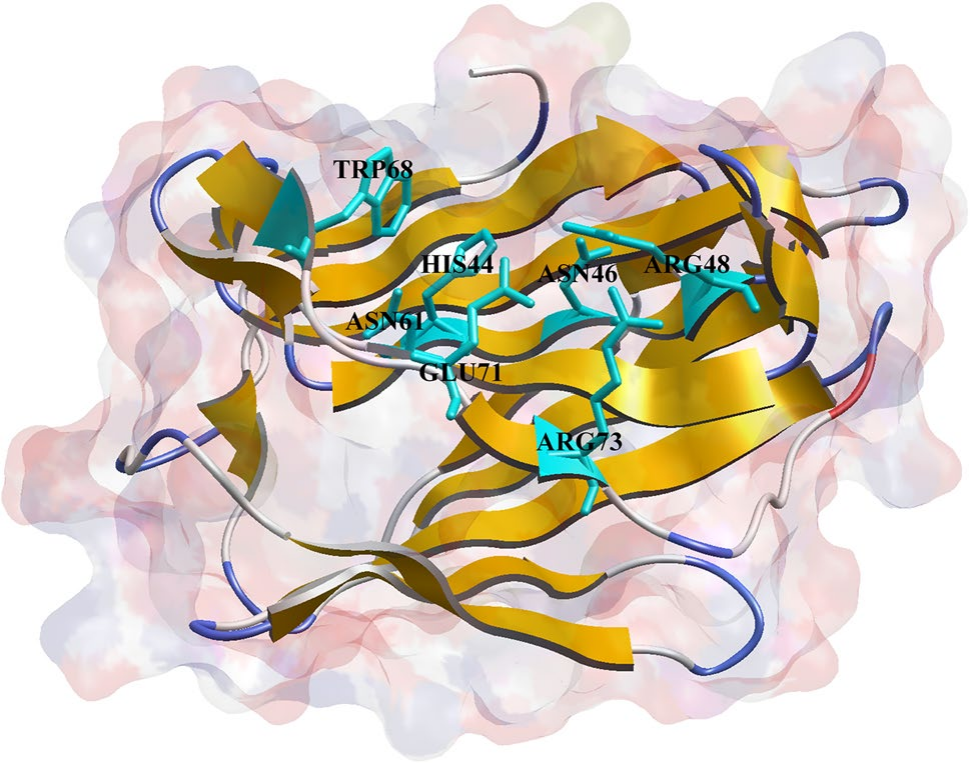
Highly conserved amino acid residues of human galectins in the CRD region; the highlighted residues develop various interactions/contacts with the carbohydrate ligands

The ‘Receptor Grid Generation’ module of the Schrödinger suite (Schrödinger, 2018) was used for generating the receptor grid file. The grid generation was accomplished using the X, Y, Z coordinates generated and selected during the DeepSite execution, confining the other close proximity residues inside the specified rectangular grid box. Particularly, the conserved residues (His44, Asn46, Arg48, Val59, Asn61, Trp68, Glu71, and Arg73) are depicted in Fig. 2, and other residues at the canonical CRD motif of galectin-1 were confined within the grid box composed of the X, Y, and Z Cartesian coordinates of 19.725, 9.673 and 16.291 Å, respectively. The grid generation was submitted in the Schrödinger suite under the default parameters due to the absence of any specific positional constraints. The successful grid generation resulted in the creation of a grid file constituting the information within the CRD region of the galectin-1 protein. The grid file was further used for the XP-docking technique.

The XP-docking protocol was performed on 56 dietary polyphenols, which were obtained using the K_DEEP_ tool-based filtration process. The 56 dietary polyphenols were docked using the prepared grid file of galectin-1 by following the standard rigid receptor docking protocol in the ‘Ligand docking’ utility tool of the Schrödinger suite [75]. The output generated a maximum of six docked poses for each polyphenol. The Glide XP docking protocol was employed in this study by following the E-model scoring function for the generation of poses, showing higher accuracy compared to the other docking protocols available for screening purposes. After successful execution of the docking procedure, each docked pose was manually inspected to investigate the interaction profile between galectin-1 and the docked conformer of each polyphenol.

### Molecular dynamics simulations and binding free energy estimations by the MM–GBSA method

The MD simulations were conducted for the five docked complexes and the apo conformation of the galectin-1 protein in order to understand the dynamic behavior of the studied protein and polyphenols under a time-dependent microcanonical ensemble. Moreover, the energetic contribution of each polyphenol for the development of several molecular binding interactions was deduced from the MD simulated trajectories. The structural behavior of the dietary polyphenols bound with the galectin-1 protein and apo structure of the protein in the dynamic states was extensively investigated by the all-atom MD simulation for a time span of 100 ns. The entire MD simulation was performed using the Amber18 [76] software package installed in the Linux platform with the 10^th^ Generation Intel Core i9–10885H and NVIDIA® GeForce RTX™ 2070. Each galectin-1 protein and polyphenol complex was immersed in a truncated octahedron of the TIP3P water model [77]. Further, the complex system was neutralized by optimizing the amounts of Na^+^ and Cl^–^ and maintaining the physiological pH during simulation. The ionic strength of the system was 0.1 M. The protein topology was generated by the ff14SB force field [78]. The MD simulation was executed at a temperature of 300 K maintained by the Langevin thermostat and a pressure of 1 atm Monte Carlo barostat with volume exchange retained. The short-range non-bonded interaction attributed to a cutoff value of 8 Å. The long-range electrostatic interactions were preserved by the particle-mesh Ewald method. A total of 10 ns equilibration was performed by employing the sequential NVT and NPT ensembles. Finally, a long-range 100 ns run was executed for the MD simulation of the protein–ligand complexes. Upon successful completion of the simulation run, various MD trajectory analyzing parameters such as root-mean-square deviation (RMSD) of the protein and ligand, root-mean-square fluctuation (RMSF), and radius of gyration (RoG) were explored using CPPTRAJ [79] over the entire MD simulated trajectory.

Further, the binding free energy of each polyphenol was obtained through the MD simulation data of the last ~ 10,000 frames of each MD simulation trajectory using the MM-GBSA method. The detailed mathematical expression of the MM-GBSA method is described in our previous publications [80, 81].

## Results and discussion

### Identification of the ligand binding site on the galectin-1 protein using DeepSite

The prediction of the specific ligand binding sites or druggable sites on the protein remains a challenge in SBDD [82–85]. The probable active binding sites on the galectin-1 protein were predicted by the DeepSite tool using a novel knowledge-based convolutional neural networks approach. In order to identify the potential drug binding site on any protein, DeepSite considers various molecular descriptors related to the protein through the 3D deep convolutional neural networks validated using an extensive test set based on over 7000 proteins from the scPDB database. Finally, the binding pocket was predicted by establishing the distance to the centers of the binding sites and comparing the discretizing volumetric overlap in order to accurately locate the chosen binding site on the galectin-1 protein.

**Fig. 3:**
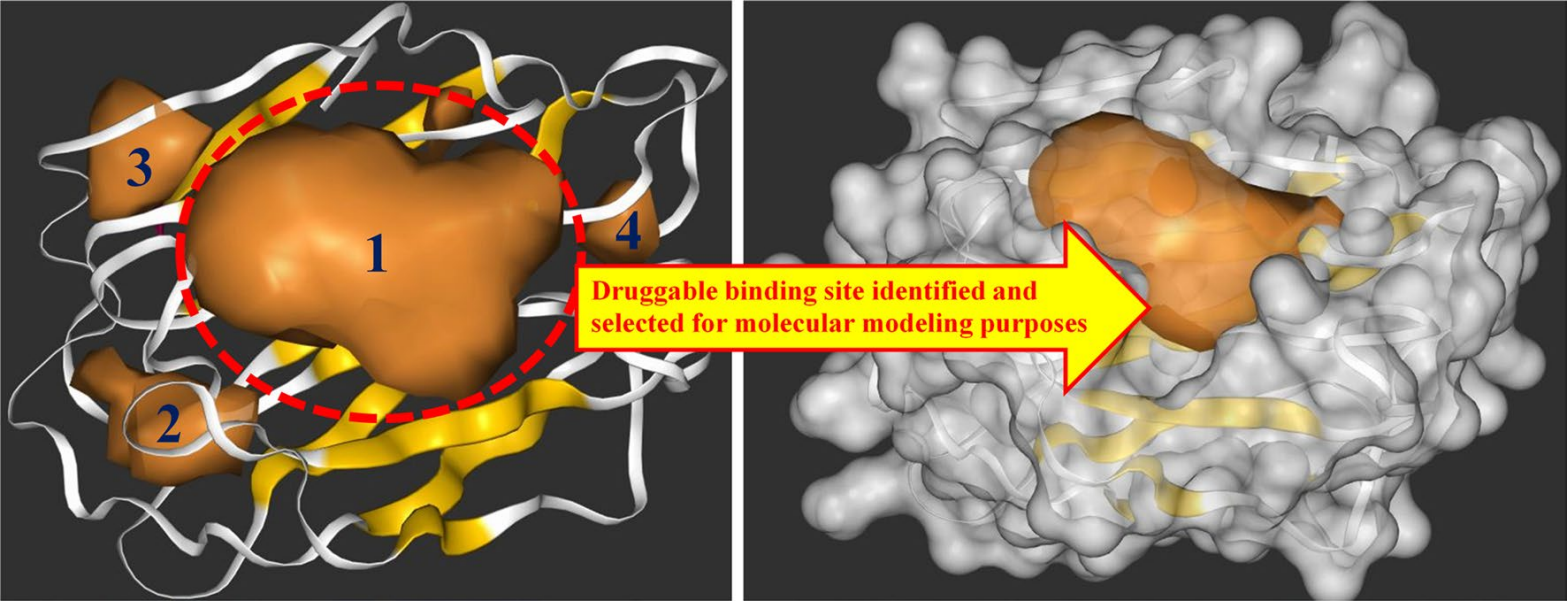
DeepSite predicted protein–ligand binding sites or druggable sites on the galectin-1 protein. Site 1 was chosen as the prominent binding site based on its highest confidence score of 0.995

The DeepSite tool predicted four (4) possible protein–ligand binding sites or druggable sites on the galectin-1 protein, as presented in Fig. 3. Interestingly, the carbohydrate binding site (CBS) region of the galectin-1 protein was found to be the most prominent druggable protein–ligand binding site by the DeepSite tool with the highest confidence score of 0.995 among the four identified binding sites. Precisely, the center coordinates of the identified site (highlighted as site 1 in Fig. 3) were described as follows: *X* = 19.725, *Y* = 9.673, and *Z* = 16.291 Å. Before further analysis of the identified binding site, the region of conserved residues or CRD motif of the galectin-1 protein was investigated to find the regions confined within the coordinates predicted by the DeepSite tool.

### Prediction of absolute binding affinity and energy of the galectin-1 protein and identified polyphenols by the K_DEEP_ program

**Fig. 4:**
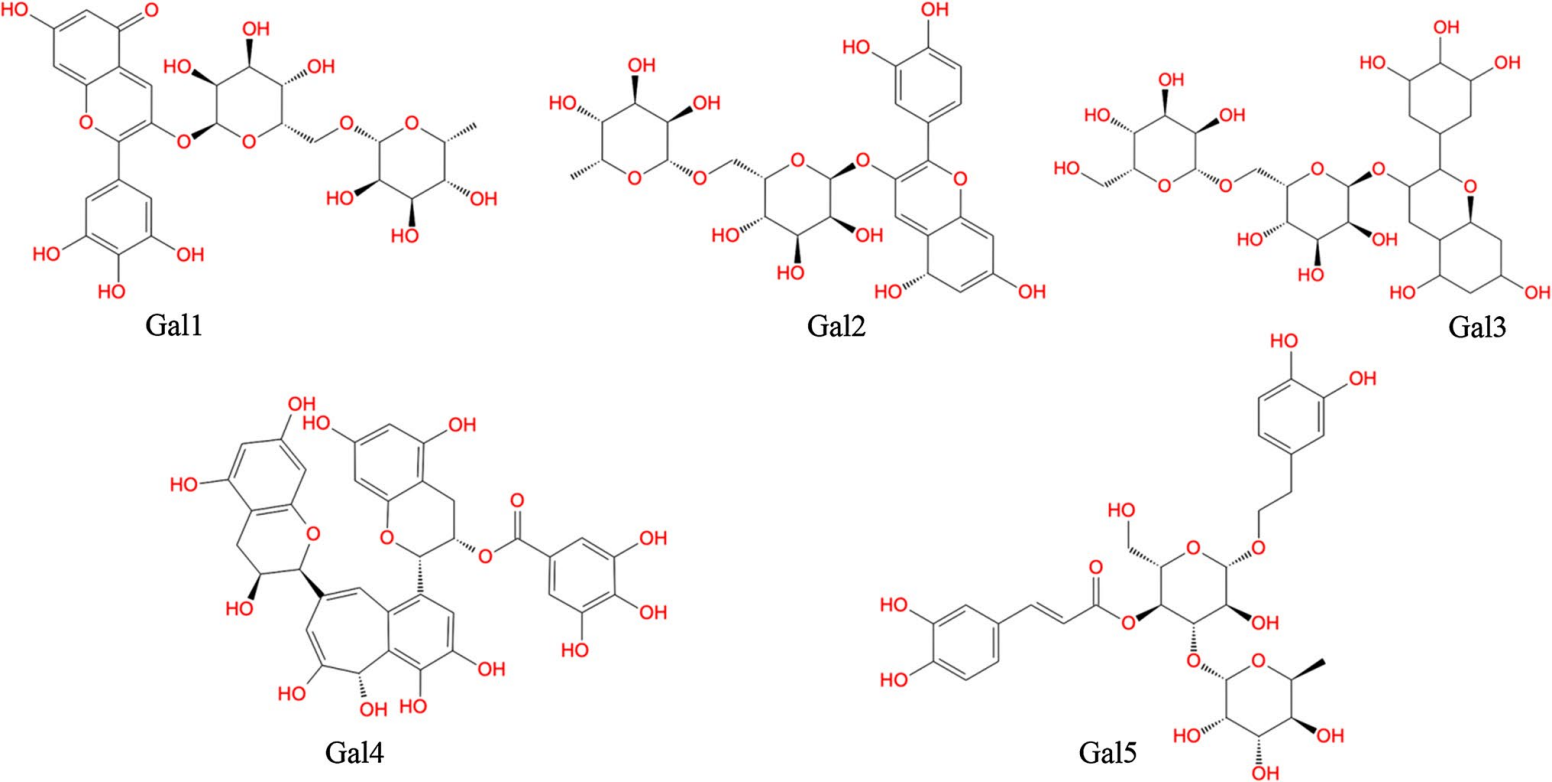
2D representation of the chemical structures of dietary polyphenols selected as potent galectin-1 protein inhibitors/modulators

In order to evaluate the absolute binding affinity and energy of each dietary polyphenol against the galectin-1 protein, the *K*_*DEEP*_ program with a 3D deep convolutional neural network-based algorithm was employed. Initially, the binding affinity and energy values of the polyphenol compounds were investigated. The binding affinity (pKd) and energy values were estimated to be in the range of 6.94 to 4.42 and –9.69 to –3.21 kcal/mol, respectively. The resultant pKd values were similar. Thus, it is difficult to differentiate the potential polyphenols against the galectin-1 protein based on the binding affinity values. However, the most highly selective dietary polyphenols binding with the galectin-1 protein was identified based on the lowest negative binding energy values of the same. This is attributed to the fact that the lowest binding energy values obtained from the *K*_*DEEP*_ program for the polyphenols corresponded to the maximum number of molecular interactions with the galectin-1 protein. The arbitrary theoretical binding energy cutoff value of –7.00 kcal/mol considered for the identification of the potential polyphenols resulted in the selection of 56 prominent dietary polyphenol compounds. The interaction potentiality of the 56 polyphenols was carefully evaluated and correlated with the extensive intermolecular interactions obtained through the molecular docking analyses. Finally, the top five dietary polyphenols were selected as strong inhibitors/modulators for galectin-1. The chemical representation of the selected polyphenols as potent galectin-1 protein inhibitor/modulator is depicted in Fig. 4.

### Glide XP docking-based binding interaction pattern and poses of the galectin-1 protein and identified polyphenol complexes

The molecular binding interactions between the galectin-1 protein and identified polyphenol complexes were obtained using the protein–ligand interaction profiler (PLIP) [86] after choosing the dietary polyphenols with the lowest docked score. Different types of molecular interactions, namely hydrophobic interactions, hydrogen bonds (H-bonds), salt bridges, π-cation, and π-stacking interactions, were observed between the galectin-1 and newly identified dietary polyphenols. The intermolecular binding interaction profiles of the five polyphenol compounds are presented in Table 1.

**Table 1:** Glide XP dock score and interacting residues of the galectin-1 protein with the five identified dietary polyphenols

Compounds	Glide XP docking score (Kcal/mol)	Interacting residues in H-bond interaction	Other types of molecular interactions
Gal1	−10.14	Ser29, Ser38, Asn46, Arg48, Asn61, Lys63, Glu71, Asp123	Lys63 (Salt bridge)/ Trp68 (π-Stacking)
Gal2	−10.46	Ser29, Asn46, Arg48, Asn61, Lys63, Glu71	Lys63 (Salt bridge)/ Val31 (Hydrophobic)/ Trp68 (π-Stacking)/ His52 (π-Cation)
Gal3	−10.94	Ser29, Asn33, Ser38, Asn39, Asn46, Lys63, Gly66, Trp68, Asp123	Trp68 (Hydrophobic)/ Lys63 (Salt bridge)
Gal4	−9.87	Asn33, His44, Arg48, Lys63, Gly66, Trp68, Asp123	Ala1 (Hydrophobic)/ Trp68 (π-Stacking)
Gal5	−9.98	Ser29, Asn46, Arg48, Lys63, Trp68, Glu71, Asp123	Leu41, Trp68 (Hydrophobic)/Lys63 (Salt bridge)

**Fig. 5:**
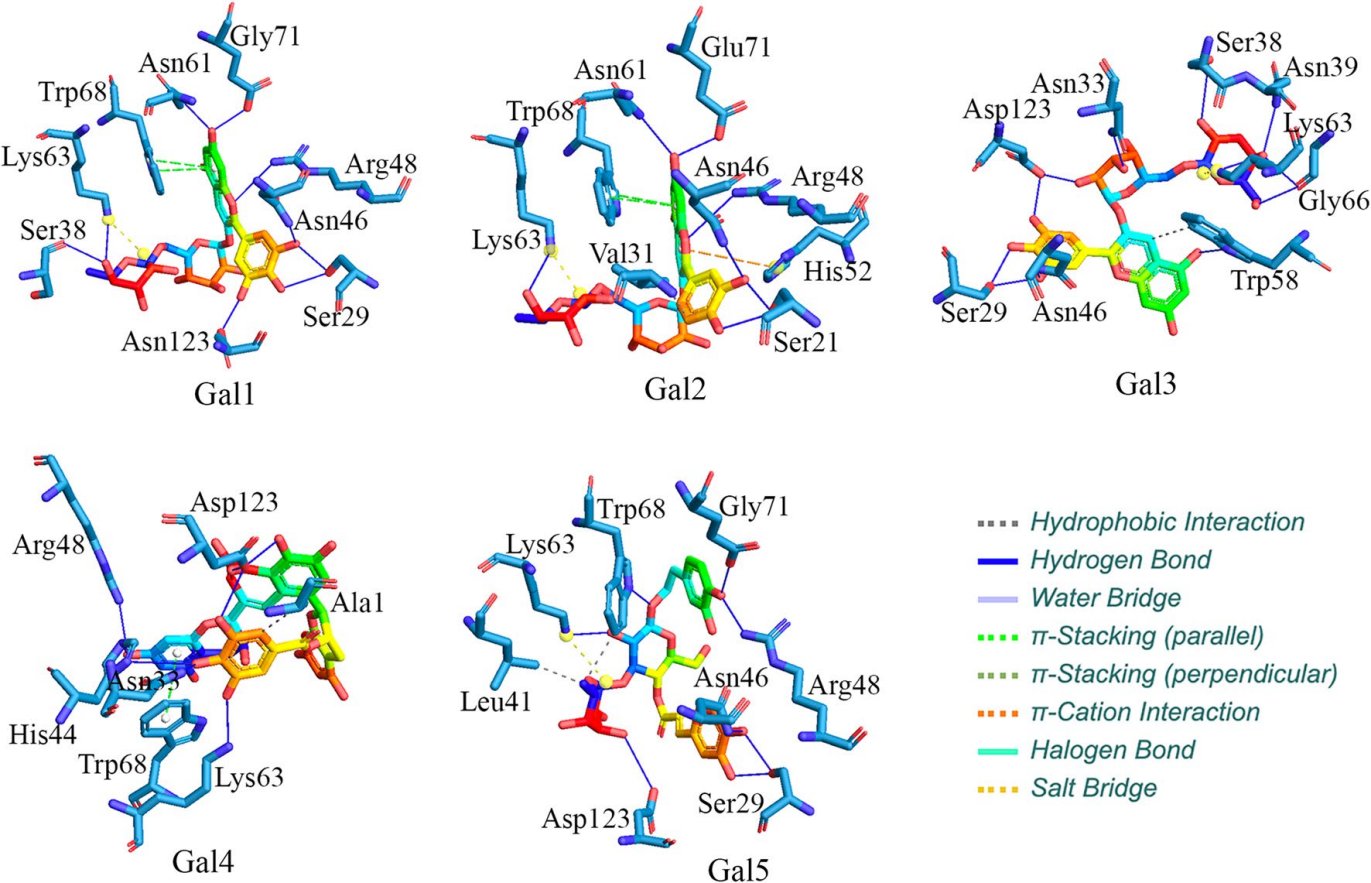
3D representation of the intermolecular binding interaction plot of the five dietary polyphenols identified as potent galectin-1 inhibitors/modulators

It was observed that the polyphenols Gal1 and Gal2 showed similar H-bonded interactions. Moreover, the Glide XP docking values of Gal1 and Gal2 were found to be –10.14 and –10.46 kcal/mol, respectively, showing obviously slight differences in magnitude. Few amino acid residues, namely Ser29, Asn46, Arg48, Asn61, Lys63, and Glu71, were found to display H-bond interactions with Gal1 and Gal2 polyphenols (Fig. 5).

**Fig. 6:**
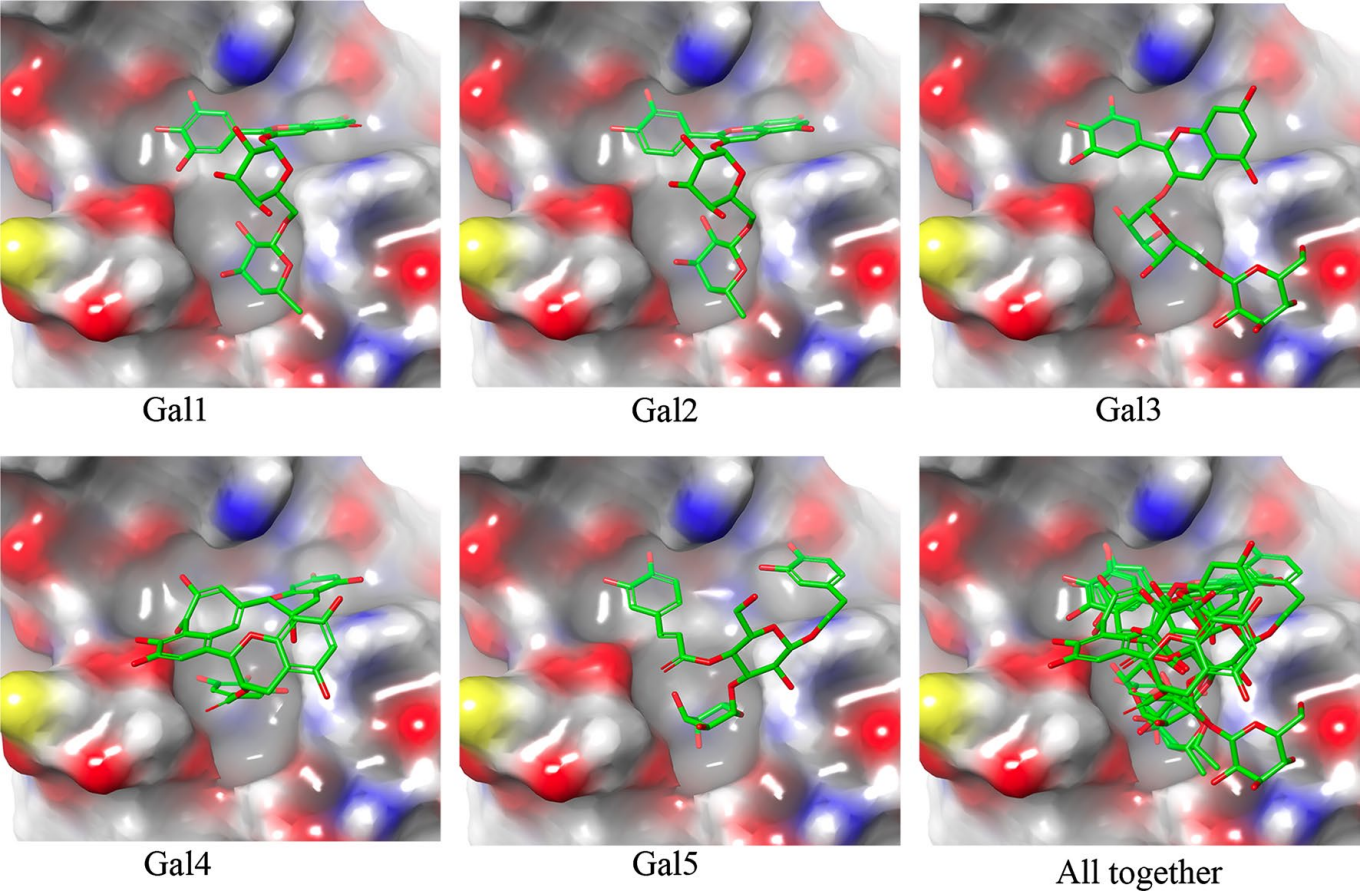
3D surface view representation of the molecular binding orientation of the galectin-1 protein with the proposed five polyphenols (Gal1, Gal2, Gal3, Gal4, and Gal5)

The H-bond lengths of the Gal1 polyphenol were in the range of 1.69 to 3.11 Å, whereas those of Gal2 polyphenol were in the range of 1.56 to 3.14 Å. The –OH group in the Gal1 and Gal2 polyphenols acted as hydrogen bond donors for the formation of H-bond interactions with the respective galectin-1 protein residues in the CRD region. The binding orientations of the two polyphenols (Gal1 and Gal2) were closely investigated and are illustrated in the surface view 3D representation in Fig. 6, revealing similar binding patterns of the compounds with the galectin-1 protein. Figure 6 suggests that all the dietary polyphenols exhibited a similar trend in the molecular interactions with the major residues existing at the deep cleft of the galectin-1 protein in the CRD region. The similar binding orientation may be attributed to their high degree of structural resemblance that influences the similar molecular conformations in the docking execution. Apart from the above-mentioned observation of the H-bonded interactions, the other two residues Lys63 and Trp68, were found to be common for the two polyphenols (Gal1 and Gal2), which exhibited salt bridge and π-stacking interactions, respectively. Another bioactive dietary polyphenol, Gal3 was structurally similar to Gal1 and Gal2 and showed similar intermolecular interaction profiles. However, some similar amino acid residues (such as Asn33, Asn39, and Gly66) were found to be involved in the formation of H-bond with Gal3. All the H-bond interaction distances in Gal3 were measured within the range of 2.02 to 3.21 Å. Although some common amino acid residues (Arg48, Lys63, Gly66, Trp68, and Asp123) were involved in the H-bond interactions of polyphenol Gal4, two other residues, namely Asn33 and His44, of the galectin-1 protein were also identified as active participants in the formation of H-bonds. Another notable observation was the occurrence of the hydrophobic contact of polyphenol Gal4 with the Ala1 residue of the galectin-1 protein. Based on the molecular docking analyses, the binding strength of polyphenol Gal4 with the galectin-1 protein was found to be relatively low, with the docking score of –9.87 kcal/mol compared to the other polyphenols. Another dietary polyphenol Gal5 showed three different types of intermolecular interactions, viz*.,* H-bond interactions, hydrophobic contact, and salt bridge interactions, with the galectin-1 protein. Gal5 was similar to the other polyphenols and showed similar H-bond interactions by the amino acid residues (Ser29, Asn46, Arg48, Lys63, Trp68, Glu71, and Asp123 of galectin-1 protein). The polyphenol showed hydrophobic interactions with the Leu41 and Trp68 residues of the galectin-1 protein and salt bridge contact with Lys63.

Overall, the binding affinities of the identified dietary polyphenols (Gal1–Gal5) in terms of the Glide XP scores showed relatively similar scoring values ranging from –10.94 to –9.87 kcal/mol. The predicted intermolecular interactions between the galectin-1 protein and identified polyphenols can be used to understand the binding selectivity and influence of specificity at the binding site in the CRD region. Specifically, the interaction of the eight conserved amino acid residues (such as His44, Asn46, Arg48, Val59, Asn61, Trp68, Glu71, and Arg73) with the five dietary polyphenols might suggest the application of the former as alternative non-carbohydrate specific binders for the galectin-1 protein.

### Comparison, influence, and importance of the binding interaction mechanisms of different amino acid residues of Galectin-1 protein in the CRD region

**Fig. 7:**
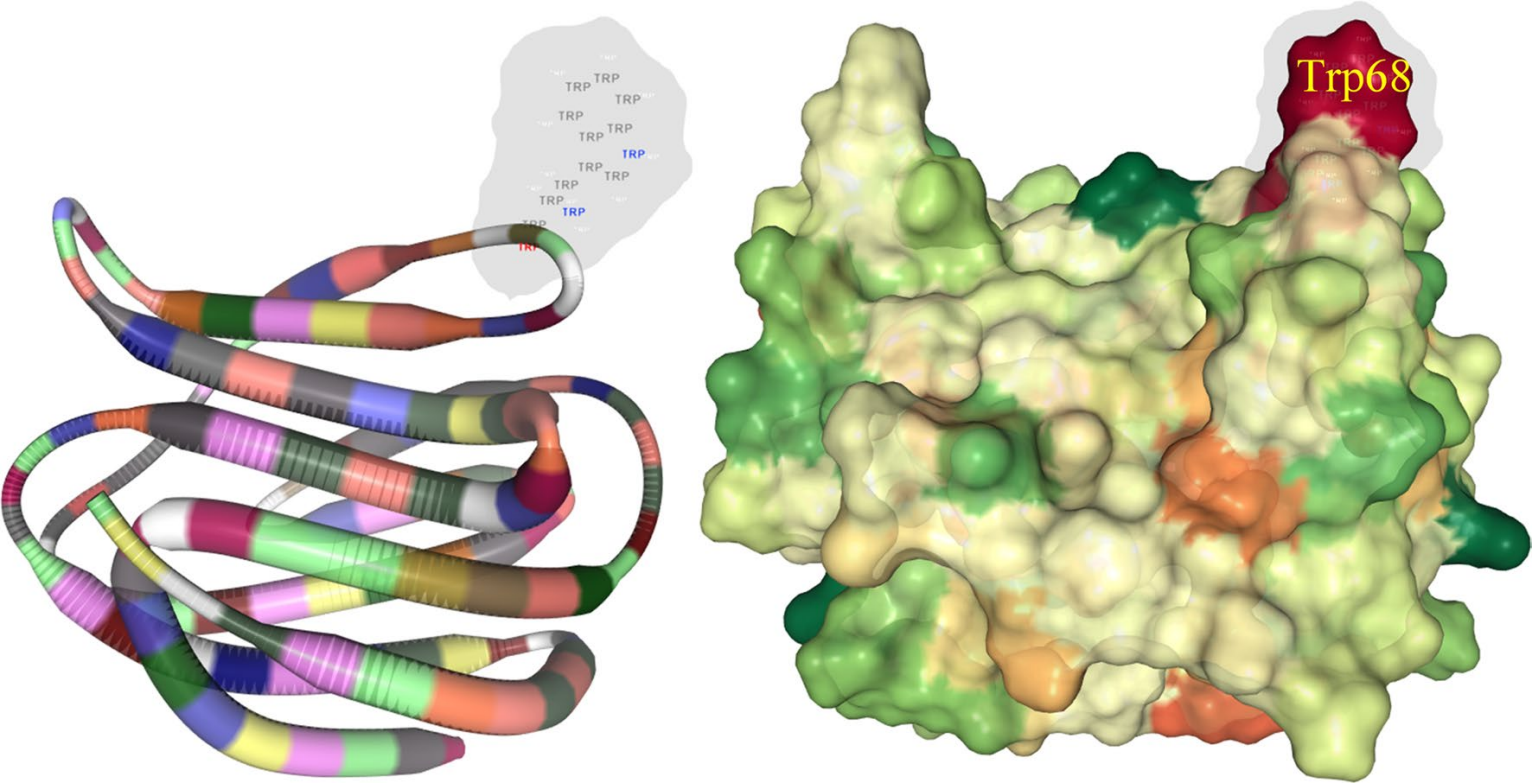
Location of the highly conserved single tryptophan amino acid residue of the galectin-1 protein in the CRD region

There are significant binding interactions between galectin-1 and dietary polyphenols, with the resultant probable mechanism being responsible for modulating the biological function of the galectin-1 protein. The obtained binding interactions were highly consistent with the previously reported results. It was observed that the highly conserved amino acid residues (such as His44, Asn46, Arg48, Val59, Asn61, Trp68, Glu71, and Arg73) of galectin-1 displayed binding interactions with most of the identified dietary polyphenols, thereby enhancing the significant binding affinity of the polyphenols in the CRD region. The CRD region of the galectin-1 protein mostly contains amino acids 44 to 71 shaped like an antiparallel β-sandwich and shows molecular binding interactions with a large series of natural ligands, including glycoproteins [87–91]. Herein, the docking-based interaction profiles of all the polyphenol compounds were found to be associated with the number of intermolecular interactions, such as H-bond, hydrophobic, π-stacking, and π-cation interactions in the deep channel of the specified residues, suggesting the crucial preference of a binding mode of the galectin-1 protein with the identified polyphenol compounds. The tryptophan (Trp68) residue depicted in Fig. 7 appears to be the only amino acid residue constituent in the CRD region of galectin-1, which is regarded as a critical amino acid due to the extensive studies reported intermolecular interaction with many compounds [92, 93]. Also, the critical role of the galectin-1 protein binding to lactose was extensively studied with respect to the specific amino residue Trp68 in the solvated galectin-1 in the ligand-free and ligand-bound states suggesting a different spectrum of tryptophan side-chain orientation [92]. Interestingly, the side-chain orientation of the Trp68 residue in the galectin-1 protein was found to be surrounded by the basic Lys63 residue with significant solvent accessibility [92]. Therefore, the critical role of the Trp68 residue might result in the formation of π-stacking, hydrophobic or H-bond interactions in the presence of any ligand, leading to the displacement of the H_2_O molecules in the surroundings of the tryptophan side chain, as observed from the results of the present study.

Moreover, many close proximity amino acid residues such as Ser62 and Thr70 of the galectin-1 protein have been reported to be crucial for establishing the water-mediated H-bond interactions with the imidazole ring of the basic amino acid residue His111 [94]. The present study also suggested that the number of H-bond interactions was significantly increased for all the dietary polyphenols found in the water environment, as discussed in the subsequent section on the MD simulation analyses. Another research on the crystallographic complex of human galectin-1 bound to LacNAc revealed some unexplored cleft on the galectin-1 protein surface constituting the amino acid Asp25 and Asp126 mediating the tailor-made ligand binding interactions [95]. In addition, the present study demonstrated the binding interactions of a few close proximity residues (Ser29 and Asp123) adjacent to the CRD region with the identified polyphenols, mimicking the binding interaction of LacNAc with galectin-1. Another recent study explored the structure–activity relationship of five-membered heterocycles of thiogalactosides and thiodigalactosides as galectin-1 inhibitors that reported the strong bond of the two compounds (thiophene 1 and thiazole 19) at the deep pocket between Ser29 and Asp123 and exhibited the selective single-digit nM affinity inhibition of galectin-1 [96]. In this study, numerous similar and comparable forms of intermolecular interactions were identified at the deep cleft between the galectin-1 residues, Ser29 and Asp123, and dietary polyphenols, which might demonstrate that the proposed compounds can also exhibit better or comparable binding inhibition affinity relative to the current common inhibitors.

Moreover, the intermolecular interactions of the bound ligand lactulose with the CRD region of the galectin-1 protein considered in this present study (PDB ID: 6B94) were investigated. Earlier, it has been demonstrated that the lactulose found to block or inhibit the galectin-1 protein, in turn, reduces lipogenesis in the adipose tissue and helps in the treatment of obesity [97]. The literature revealed the existence of some conserved contacts with the CRD region formed by the galactose ring of lactulose [59]. Precisely, the study reported that the galactose unit interacts with His52 and Trp68 via stacking, while the C4-OH group of the galactose interacts with the amino acid residues His44, Asn46, and Arg48 via hydrogen bonding interactions. The C6-OH group has also formed H-bonds mediated by the amino acid residues, namely Asn61 and Glu71, whereas the C5-OH group stabilized by the formation of contacts with the amino acid residues Arg48 and Glu71. Also, it has been reported that the basic amino acid residue His52 interacts with the C2-OH group of the galactose ring. Interestingly, the involvement of the fructose ring in the establishment of the two H-bonded interactions between the amino acid residues Arg73 and C1'-OH, and Glu71 and C2'-OH has significantly enhanced the binding affinity of lactulose with galectin-1 [59]. Undoubtedly, the reported intermolecular interactions of different inhibitor molecules with important amino acid residues of the galectin-1 protein have determined their inhibition efficacy at different levels. In the present study, the identified dietary polyphenols (Gal1–Gal5) showed similar types of intermolecular interactions, as reported in the literature. In addition, the study reported additional contact formations that might lead to similar or better inhibition efficacy toward the galectin-1 protein on binding with the proposed polyphenols.

### Molecular dynamic simulation analyses of the identified polyphenols and galectin-1 complexes

The MD simulation approach is widely used to explore the dynamic behavior of the macromolecules or macromolecule-small molecule complexes. It is a process of computational simulations that numerically integrates the equations of motion of atoms and molecules. The MD simulation is an excellent method to explore the stability of the macromolecule as well as the macromolecule-small molecule associations. In the current study, the best-docked complexes formed between the proposed molecules and galectin-1 were taken into consideration for the 100 ns conventional MD simulation study. Several parameters, namely the protein backbone RMSD, RMSF, ligand atoms RMSD, RoG, total hydrogen bonds, and SASA, were estimated from the MD simulation trajectory. Moreover, the binding affinity of each molecule toward the galectin-1 protein was calculated using the MM–GBSA approach. For better insight, the average, maximum and minimum values of RMSD, RMSF, RoG, and SASA were calculated and are tabulated in Table 2.

**Table 2:** Details of the protein backbone RMSDs, ligand RMSDs, RMSFs, RoG, and SASA values obtained from the MD simulation trajectory analyses

	Apo	Gal1	Gal2	Gal3	Gal4	Gal5
*RMSD (Å)*						
Average	1.198	1.387	1.000	1.477	1.095	1.074
Maximum	1.914	2.099	2.241	2.147	1.965	1.534
Minimum	0.426	0.537	0.498	0.538	0.524	0.514
*Ligand-RMSD (Å)*						
Average	–	1.775	1.662	3.376	1.443	2.385
Maximum	–	4.420	4.550	5.159	3.435	3.633
Minimum	–	0.000	0.000	0.000	0.000	0.000
*RMSF (Å)*						
Average	10.299	10.253	9.749	9.922	9.922	9.155
Maximum	17.672	16.525	15.652	16.617	16.617	14.656
Minimum	3.724	2.813	2.864	2.716	2.716	3.280
*Radius of gyration (Å)*						
Average	14.024	13.994	13.985	13.972	13.986	13.962
Maximum	14.264	14.262	14.230	14.207	14.194	14.167
Minimum	13.814	13.783	13.763	13.765	13.781	13.792
*Solvent-Accessible Surface Area (Å*^*2*^*)*						
Average	6688.697	6371.677	6459.572	6391.728	6448.650	6188.651
Maximum	7413.535	7174.001	7301.842	7288.672	7182.663	6874.287
Minimum	5960.536	5537.560	5756.433	5706.685	5794.141	5614.324

### Root-mean-square deviation

**Fig. 8:**
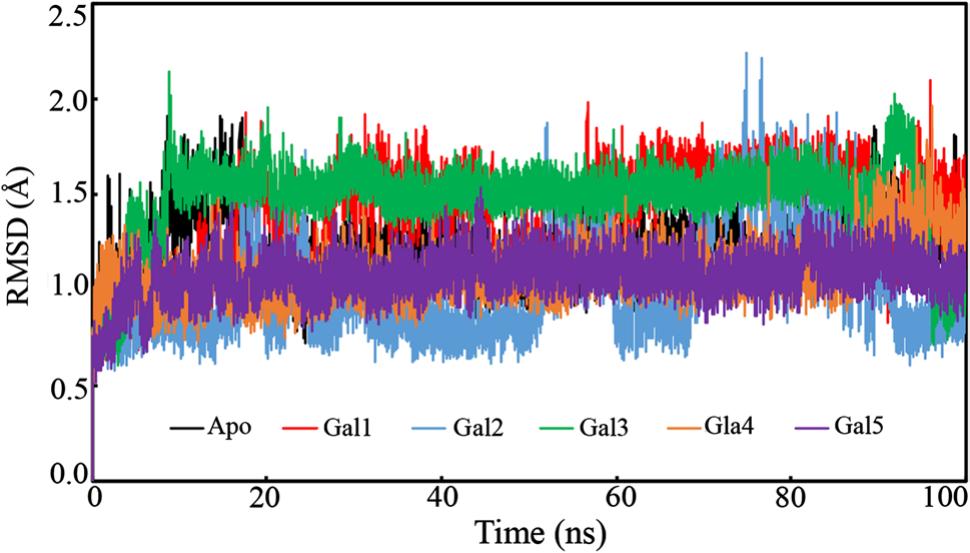
The RMSD plot of the apo structure of the galectin-1 protein backbone and galectin-1 bound with the polyphenols Gal1, Gal2, Gal3, Gal4, and Gal5

The protein backbone RMSD is one of the significant parameters of the MD simulation trajectory used to explore the backbone deviation of an individual frame generated in a dynamic environment. The unfolding of the protein molecule generates a high value of RMSD and conversely indicates the compactness. The consistent variation in the RMSD value over the period of simulation signifies the equilibration of the protein–ligand complex. The backbone of each protein frame was considered for the RMSD calculation, followed by the plotting of these values against the time of simulation, as shown in Fig. 8. Also, the apo-galectin-1 protein was used for the simulation in order to compare the deviation of the galectin-1 backbone upon binding with different small molecules. It was observed that the backbone of the galectin-1 protein bound to the proposed molecules showed a similar deviation as that of the apo-galectin-1 protein with a slight variation. The RMSD values of the complexes increased up to 5 ns and then remained consistent until the end of the simulation. It was observed that the galectin-1 backbone bound with Gal4 was equilibrated with a lower RMSD value compared to the apo-galectin-1 protein. Although the galectin-1 backbone bound with Gal1 and Gal3 showed a slightly higher RMSD value, the variation pattern clearly explained the consistency throughout the simulation. The Gal2 bound galectin-1 backbone was found to deviate frequently in comparison to others, but the deviation was less than 2.3 Å. Moreover, such frequent deviation might be attributed to the opening and closing of the protein to accommodate Gal2 perfectly inside the binding pocket. The average RMSD value indicates the deviation of the galectin-1 backbone during the simulation period. The average RMSD values of the galectin-1 backbone bound to Gal1, Gal2, Gal3, Gal4, and Gal5 were found to be 1.387, 1.000, 1.477, 1.095, and 1.074 Å, respectively. The average RMSD value of the apo-protein backbone was found to be 1.198 Å. The above RMSD data and outline of the variation during the simulation explained the stability of the protein and small molecules.

**Fig. 9:**
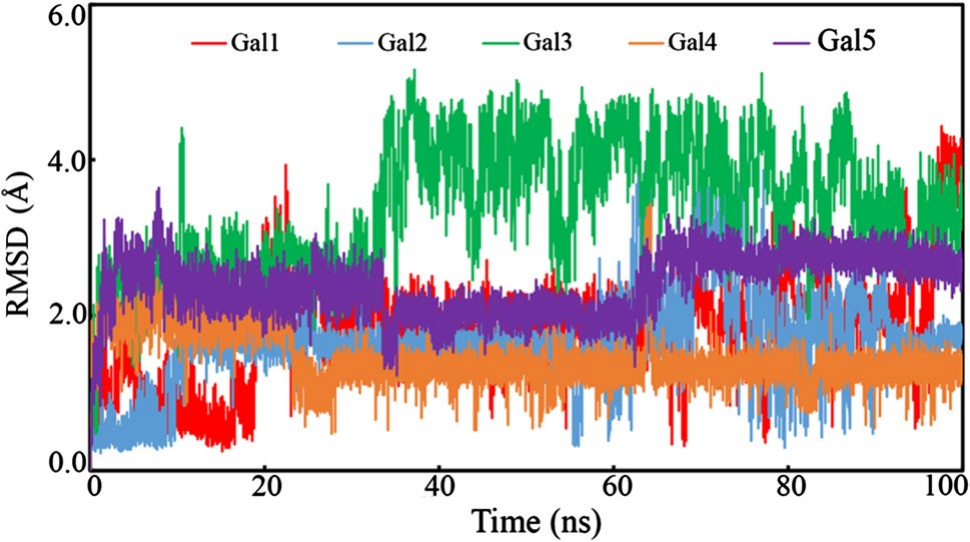
The RMSD of the individual polyphenol compounds, Gal1, Gal2, Gal3, Gal4, and Gal5, over 100 ns span of the MD simulation

It is important to explore the deviation of the bound small molecules inside the receptor during the simulation. The RMSD value over the time of simulation for the proposed molecules was calculated, as given in Fig. 9. Except for Gal3, all the molecules showed consistent changes in the RMSD outline. Up to 35 ns, the RMSD value of Gal3 was found to deviate in a similar pattern as that of the other polyphenols, which later increased to about 4 Å and equilibrated till the end of the simulation. This change in the deviation might be attributed to the conformational change in the molecule. The difference between the highest and average values can give insight into the variation of the molecules in the dynamic states. From Table 2, the difference between the maximum and average RMSD values of Gal1, Gal2, Gal3, Gal4, and Gal5 was found to be 2.645, 2.888, 1.783, 1.992, and 1.248 Å, respectively. The above observations explained the compactness of the small molecules along with few variations during the simulation.

### Root-mean-square fluctuation

**Fig. 10:**
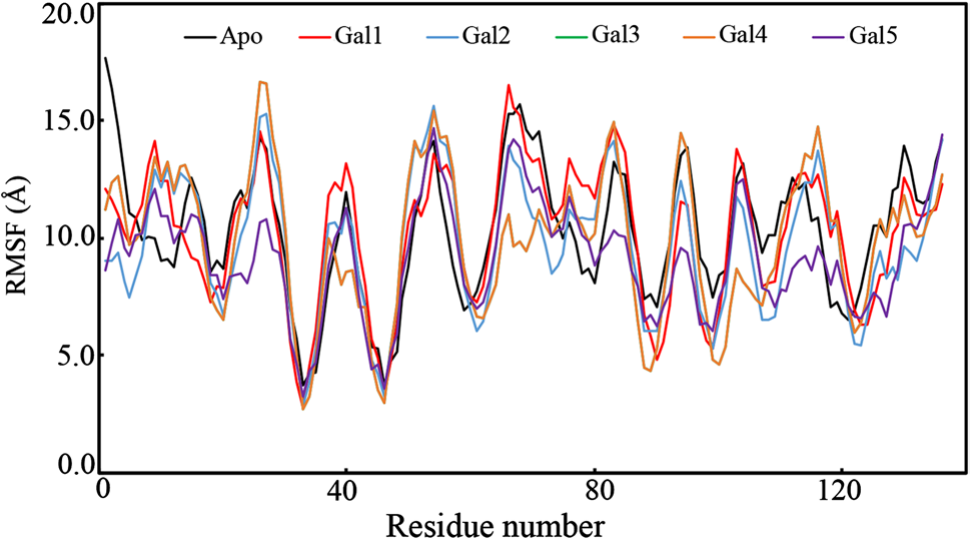
The RMSF of the amino acid residues of apo-galectin-1 and galectin-1 bound with the dietary polyphenol compounds Gal1, Gal2, Gal3, Gal4, and Gal5

In the MD simulation experiment, the individual amino acid residue plays a crucial role in the stability of the protein–ligand complex. The residue fluctuation during the simulation can be explored by the RMSF parameter. From the MD simulation trajectory, the RMSF value was calculated and is shown in Fig. 10. It is very interesting to note that the amino residues of galectin-1 bound with Gal1, Gal2, Gal3, Gal4, and Gal5 fluctuated in a similar manner as that of apo-galectin-1. The above observation indicates the slight deviation of the amino residues due to the incorporation of small molecules inside the receptor site. The average RMSF was found to be 10.299, 10.253, 9.749, 9.922, 9.22, and 9.155 Å for the apo-galectin-1 structure and galectin-1 protein bound with the polyphenols Gal1, Gal2, Gal3, Gal4, and Gal5, respectively.

### Radius of gyration

**Fig. 11:**
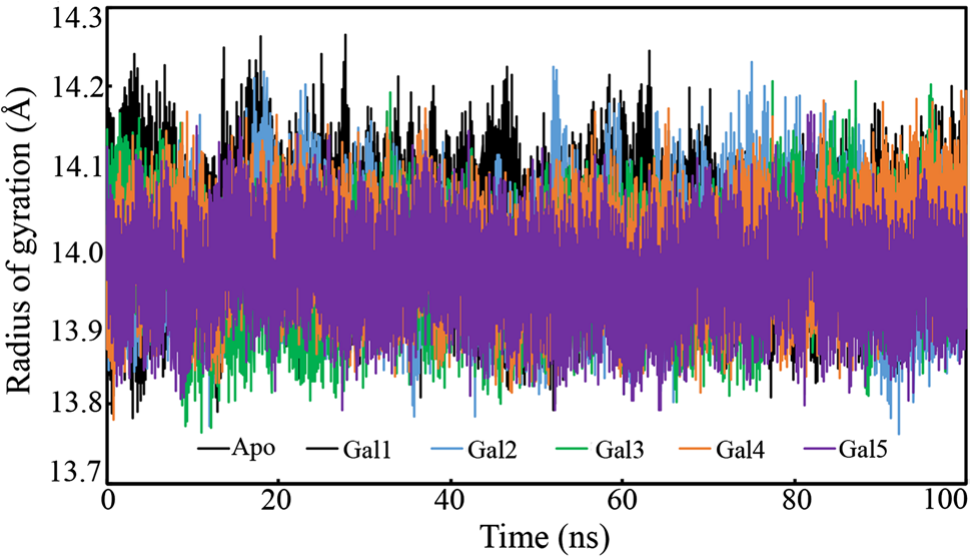
The radius of gyration of galectin-1 in the apo form and bound with the polyphenols Gal1, Gal2, Gal3, Gal4, and Gal5

The rigidity and compactness of the macromolecule during the MD simulation can be assessed from its RoG. A high value or abnormal variation of RoG in different frames signifies the instability of the macromolecule, whereas a low and consistent variation of RoG indicates the stable folding of the protein. In order to explore the folding and unfolding pattern of galectin-1 bound with the proposed molecules, the RoG was calculated from each MD simulation trajectory. The RoG of apo-galectin-1, galectin-1 bound with Gal1, Gal2, Gal3, Gal4, and Gal5 were plotted against the time of simulation, as shown in Fig. 11. It was found that the RoG of all the systems was in the range of 17.75 to 14.25 Å. The galectin-1 protein in the apo form and bound to the proposed molecules showed a uniform equilibrated variation in RoG during the MD simulation experiment. There was no abnormal deviation in any of the systems. The range of variation can be extracted by the calculation of the difference between the maximum and minimum RoG values of each system. The difference between the maximum and minimum RoG values of apo-galectin-1, galectin-1 bound with Gal1, Gal2, Gal3, Gal4, and Gal5 was found to be 0.450, 0.479, 0.467, 0.442, 0.413, and 0.375 Å, respectively. The low range of deviation and consistency in the variation of RoG undoubtedly explained the compactness of galectin-1 during the simulation in the apo form and as a complex with the proposed molecules.

### Analysis of the intermolecular hydrogen bond

**Fig. 12:**
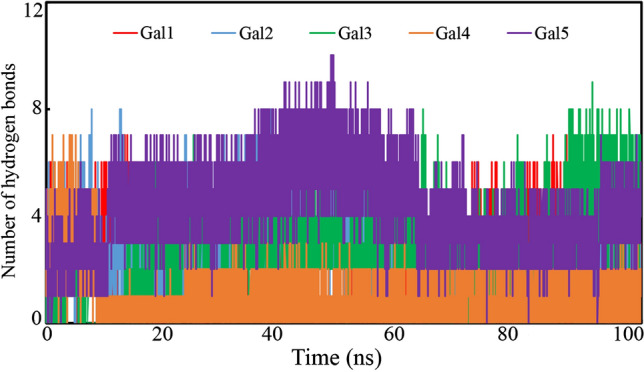
Number of hydrogen bonds formed between galectin-1 and proposed inhibitors/modulators

The hydrogen bond between the small molecule and ligand-binding amino acid residues firmly holds the complex and offers stability. The hydrogen bonds between the proposed molecules and amino acid residues present on the active site of galectin-1 were calculated and are displayed in Fig. 12. A few frames were found to form a large number of hydrogen bonds and strongly hold the protein–ligand complex. Except for Gal4, all the proposed molecules were found to form a large number of hydrogen bonds in the maximum number of frames. The hydrogen bond formation was found to be ranging from 0 to 10, which clearly explained the strong association between galectin-1 and proposed molecules. Hence, the large number of hydrogen bonds gave extra strength to retain the molecules inside the receptor cavity of galectin-1.

### Solvent-Accessible Surface Area (SASA)

**Fig. 13:**
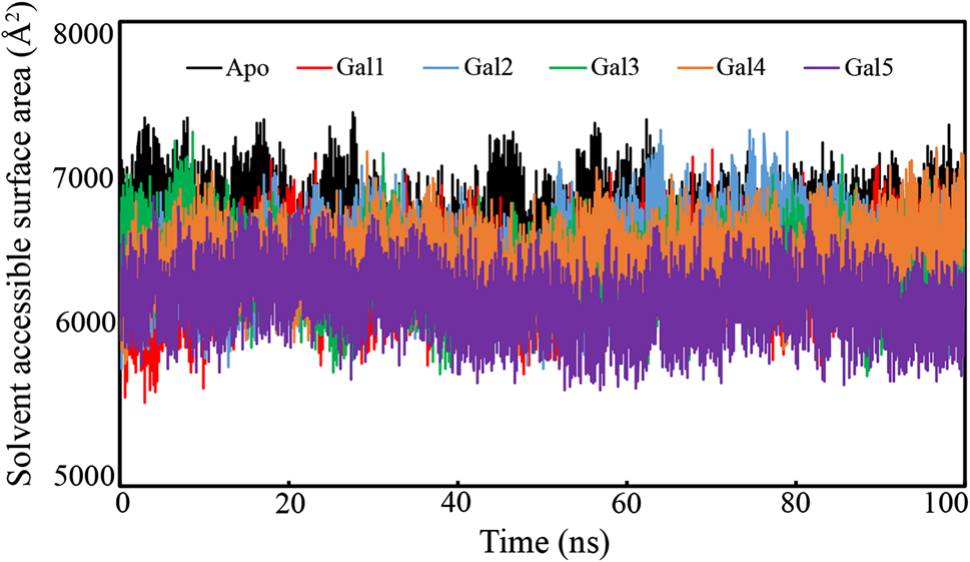
The solvent-accessible surface area of galectin-1 in the apo form and galectin-1 bound with the polyphenols Gal1, Gal2, Gal3, Gal4, and Gal5

The SASA of a biomolecular system measures the surface area accessible to the solvent. It is important to form the interactions between the macromolecule and solvent used in the MD simulation study. A time-dependent SASA of apo-galectin-1 and galectin-1 bound with Gal1, Gal2, Gal3, Gal4, and Gal5 was calculated, as presented in Fig. 13. It is crucial to note that there is no significant variation in the SASA values in any of the galectin-1 proteins bound to the proposed molecules. The average SASA values of galectin-1 in the apo form, galectin-1 bound to Gal1, Gal2, Gal3, Gal4, and Gal5 were found to be 6688.697, 6371.677, 6459.572, 6391.728, 6448.650, and 6188.651 Å^2^, respectively. The stable and relatively similar SASA values observed for the identified dietary polyphenols during the MD simulations indicate the consistent hydrophobic interactions between the nonpolar residues during the protein folding process in the dynamic state. However, the SASA values for the apo-galectin-1 protein system revealed a slight increase or some degree of fluctuation at a certain time period, suggesting the enhanced solvation of the hydrophobic core with the increase in the folding process. This may be attributed to the presence of any unbound ligand with the apo-galectin-1 molecular system.

### Estimation of the binding free energy using the MM-GBSA approach

The MM-GBSA method is widely used to calculate the binding free energy of a small molecule toward the receptor. This approach uses the MD simulation trajectories of the receptor–ligand complex to estimate the binding free energy and thus offers accuracy and computational effectiveness in comparison to the empirical scoring and strict alchemical perturbation methods [98]. Using the above-mentioned approach, the binding free energy of the proposed molecules was estimated, as presented in Table 3. The Gal5 polyphenol showed the strongest affinity toward the galectin-1 protein with the binding free energy of –39.040 kcal/mol. The binding free energy of Gal2 and Gal3 was similar with the values of –38.548 and –36.552 kcal/mol, respectively. The binding free energy of the dietary polyphenols Gal1 and Gal4 toward galectin-1 was found to be –31.564 and –33.228 kcal/mol, respectively. Hence, all the molecules were found to have a significant binding affinity toward the galectin-1 protein, which might be crucial inhibitors with some optimization characteristics.

**Table 3:** Average binding free energy of the dietary polyphenols Gal1, Gal2, Gal3, Gal4, and Gal5 bound with galectin-1estimated from the MD simulation trajectories

Identified polyphenols	Energy (Kcal/mol)		
	Elec^1^	vdW^2^	*∆G*_*bind*_
Gal1	− 48.806	− 26.420	− 31.564
Gal2	− 13.840	− 11.218	− 38.548
Gal3	− 24.962	− 7.534	− 36.552
Gal4	− 32.456	− 16.749	− 33.228
Gal5	− 66.386	− 18.128	− 39.040

^1^Electrostatic; ^2^van der Waal’s

## Conclusion

In the present study, an exhaustive computational approach was adopted for the identification and establishment of the interaction mechanism of the potential dietary polyphenols for galectin-1 inhibition/modulation. The study revealed that the CRD region of the galectin-1 protein could be the most prominent ligand binding site for targeting the prototype-specific galectin. Moreover, the DCNN algorithm-based application estimated highly negative binding affinities and energies for the selected dietary polyphenols, suggesting a strong interaction potentiality for the selected compounds toward the galectin-1 protein. Also, a majority of the molecular binding interactions were formed by the conserved amino acid residues, such as His44, Asn46, Arg48, Val59, Asn61, Trp68, Glu71, and Arg73, of the galectin protein family, suggesting the ability of the identified polyphenols to specifically interfere the CRD region of galectin-1 to bring out some degree of effective biological regulation for the modulation of the galectin-1 function. It was observed that the single amino acid residue tryptophan (Trp68) present in the galectin-1 protein affected its intermolecular interaction in the hydrophilic or hydrophobic environment. Since this residue constitutes the galactoside-binding pocket, any molecular interaction with this aromatic residue and small molecules will certainly provide some level of biological impact on the inhibition or modulation of the galectin-1 protein. The present study revealed that most of the proposed dietary polyphenols critically interacted with Trp68 on the galectin-1 protein mediating through several intermolecular interactions. Furthermore, dynamic stability and binding free energies of each dietary polyphenol affirmed stable and strong conformational integrity with the galectin-1 protein, as observed from the extensive long-range MD simulation study and MM-GBSA-based binding free energy estimation. Nonetheless, the results suggested that the galectin-1 protein can be targeted through other non-carbohydrate ligands with better specificity. Therefore, the present study provided significant insight into the new opportunities for further exploitation of the dietary polyphenols as specific inhibitors/modulators of galectin-1 or other members of the galectin family. However, extensive experimental validation through in vivo or in vitro settings is required for gaining a better understanding of the involved mechanism.
